# Pan-genome wide association study of *Glaesserella parasuis* highlights genes associated with virulence and biofilm formation

**DOI:** 10.3389/fmicb.2023.1160433

**Published:** 2023-04-17

**Authors:** You Zhou, Dike Jiang, Xueping Yao, Yan Luo, Zexiao Yang, Meishen Ren, Ge Zhang, Yuanyuan Yu, Aiping Lu, Yin Wang

**Affiliations:** ^1^Key Laboratory of Animal Diseases and Human Health of Sichuan Province, College of Veterinary Medicine, Sichuan Agricultural University, Chengdu, China; ^2^Law Sau Fai Institute for Advancing Translational Medicine in Bone and Joint Diseases (TMBJ), School of Chinese Medicine, Hong Kong Baptist University, Kowloon Tong, Hong Kong SAR, China; ^3^Guangdong-Hong Kong-Macao Greater Bay Area International Research Platform for Aptamer-Based Translational Medicine and Drug Discovery (HKAP), Hong Kong, Hong Kong SAR, China; ^4^Institute of Integrated Bioinformedicine and Translational Science (IBTS), School of Chinese Medicine, Hong Kong Baptist University, Kowloon Tong, Hong Kong SAR, China

**Keywords:** *Glaesserella parasuis*, pan-genome, GWAS, the core genome, the accessory genome, virulence, biofilm formation

## Abstract

*Glaesserella parasuis* is a gram-negative bacterium that causes fibrotic polyserositis and arthritis in pig, significantly affecting the pig industry. The pan-genome of *G. parasuis* is open. As the number of genes increases, the core and accessory genomes may show more pronounced differences. The genes associated with virulence and biofilm formation are also still unclear due to the diversity of *G. parasuis*. Therefore, we have applied a pan-genome-wide association study (Pan-GWAS) to 121 strains *G. parasuis*. Our analysis revealed that the core genome consists of 1,133 genes associated with the cytoskeleton, virulence, and basic biological processes. The accessory genome is highly variable and is a major cause of genetic diversity in *G. parasuis*. Furthermore, two biologically important traits (virulence, biofilm formation) of *G. parasuis* were studied *via* pan-GWAS to search for genes associated with the traits. A total of 142 genes were associated with strong virulence traits. By affecting metabolic pathways and capturing the host nutrients, these genes are involved in signal pathways and virulence factors, which are beneficial for bacterial survival and biofilm formation. This research lays the foundation for further studies on virulence and biofilm formation and provides potential new drug and vaccine targets against *G. parasuis*.

## 1. Introduction

*Glaesserella parasuis* (formerly known as Haemophilus parasuis) is the etiologic agent of Glässer’s disease in pigs. Glässer’s disease is characterized by fibrinous polyserositis and meningitis (Oliveira and Pijoan, 2004). Glässer’s disease is an important infectious disease in the world pig industry (Sun et al., 2022). Glässer’s disease is not only the main infectious disease of nursery pigs but also affects the health of fattening pigs and sows. *G. parasuis* has been reported to bring substantial economic losses to the pig industry in various countries and is the third most crucial bacterial pathogen affecting finisher herds (Rivera-Benítez et al., 2021).

The pan-genome is the assemblage of all the genes in a given species (Barh et al., 2020; Sherman and Salzberg, 2020; Her et al., 2021). Generally, the pan-genome consists of the core genome and the accessory genome. The boundaries of the core genome can be extrapolated from highly conserved genes (Iranzadeh and Mulder, 2019). Importantly, the core genome is the backbones of all strains of a specie (Segerman, 2012). The accessory genome assemblages of genes unique to different individuals (Kung et al., 2010). The accessory genome reflects bacteria resistance and adaptation to different environmental conditions (Jackson et al., 2011). Moreover, some studies have reported that the pan-genome of *G. parasuis* is open (Li et al., 2013). The openness of *G. parasuis* pan-genome reflects the diversity of the gene pool among the strains of *G. parasuis* (Bentley, 2009). Open pan-genome is the main reason for the low cross-protection of existing vaccines. As the number of genes increases, the core genome and the accessory genome may show further differences in virulence, function, metabolic pathways, etc. However, the specific differences between the core genome and the accessory genome are uncertain in *G. parasuis*.

To date, *G. parasuis* can be divided into 15 serovars *via* a gel immuno-diffusion assay. Moreover, a molecular serotyping method is established according to the polysaccharide capsule of the standard strains (Nedbalcova et al., 2006). Generally, some serovars, such as 4, 5, and 13, are more pathogenic than others (Rapp-Gabrielson and Gabrielson, 1992). However, specific serovars previously considered nontoxic can still exhibit pathogenicity. These cases may reveal no absolute relationship between virulence and serovars (Aragon et al., 2010). In clinical cases, some clinical symptoms presenting as acute or chronic may result from the virulence of different strains. Although the genome of *G. parasuis* has long been obtained, the function and essentiality of the annotated genes remain largely unknown, especially virulence factors (Costa-Hurtado and Aragon, 2013; Zhang et al., 2014). In pathogenic bacteria, virulence factors play a critical role in pathogenesis. For example, the VtaA8 and VtaA9 proteins, belonging to the member of trimeric autotransporters, have phagocytosis resistance to evade the host innate immune responses (Costa-Hurtado et al., 2012). However, only very few virulence factors were identified in previous studies. The role of most virulence factors in adhesion to and invasion of host cells, resistance to phagocytosis by macrophages, resistance to serum complement, and induction of inflammation remains uncertain.

Biofilm formation is central to promoting colonization, changing strains’ adhesion and even increasing drug resistance (Watnick and Kolter, 2000; Lewis, 2001; Kokare et al., 2009). Moreover, biofilm formation also plays an essential role in the phenotype switch from commensal to pathogen. In previous studies, *G. parasuis* had the ability to form biofilm (Jin et al., 2006). The part gene expression levels of *G. parasuis* changed significantly before and after the formation of the biofilm (Jiang R. et al., 2021). For instance, the cytoplasmic heme-binding protein HutX, characterized by transporting the heme-degrading enzyme HutZ, can affect the formation of the biofilm and result in chronic infection of *G. parasuis* (Sekine et al., 2016). This may provide a clue to the pathogenic mechanism. Glycogen operon protein GlgX, playing an essential role in starch and sucrose metabolism pathways, can maintain the structural integrity of the biofilm, suggesting that metabolism-related genes are similarly closely related to biofilm formation (Sheppard and Howell, 2016; Jiang R. et al., 2021). However, these genes related to biofilm formation have still been explored.

In the present study, we reported a pan-genome-wide association study of genome sequence from 121 *G. parasuis* natural population isolates. This study revealed the major difference between the core genome and the accessory genome. Our results highlight that these genes with causal relationships to virulence and biofilm formation capacity were deeply excavated in the association of traits and genes.

## 2. Materials and methods

### 2.1. Bacterial strains

In this study, the complete genomes of 105 *G. parasuis* strains with geographical, virulent and serological diversity were retrieved. These strains were available in March 2020 from NCBI.^1^ Information about the 105 strains is summarized in Supplementary Table S1.

Additionally, 16 clinical strains isolated in Sichuan between 2015 and 2020 were sequenced in our previous study. Information about the 16 strains were available from China National GeneBank DataBase (CNGBdb).^2^ Project number is CNP0002150. Sequencing information about the 16 strains is summarized in Supplementary Table S2. And the background information of *G. parasuis* isolates showed in Table 1.

**Table 1:** The background information of *G. parasuis* isolates.

Name	Species identification	Isolated regions	Serotype	Isolated area (Sichuan Province)
GP 01	*Glaesserella parasuis*	Lung lesion tissue	5	Suining city
GP 02	*Glaesserella parasuis*	Lung lesion tissue	5	Mianyang city
GP 03	*Glaesserella parasuis*	Lung lesion tissue	5	Suining city
GP 04	*Glaesserella parasuis*	Lung lesion tissue	10	Nanchong city
GP 05	*Glaesserella parasuis*	Lung lesion tissue	1	Pujiang county
GP 06	*Glaesserella parasuis*	Lung lesion tissue	5	Pujiang county
GP 07	*Glaesserella parasuis*	Joint tissue effusion	Unknow	Nanchong city
GP 08	*Glaesserella parasuis*	Lung lesion tissue	5	Suining city
GP 09	*Glaesserella parasuis*	Lung lesion tissue	5	Pujiang county
GP 10	*Glaesserella parasuis*	Joint tissue effusion	Unknow	Nanchong city
GP 11	*Glaesserella parasuis*	Lung lesion tissue	7	Guangan city
GP 12	*Glaesserella parasuis*	Lung lesion tissue	Unknow	Suining city
GP 13	*Glaesserella parasuis*	Lung lesion tissue	10	Suining city
GP 14	*Glaesserella parasuis*	Lung lesion tissue	7	Xichang city
GP 15	*Glaesserella parasuis*	Joint tissue effusion	unknow	Pi county
GP 16	*Glaesserella parasuis*	Lung lesion tissue	5	Suining city

### 2.2. Pan-genomic construction

To sustain the consistency and reliability of gene prediction and annotation, the Prokaryotic Genome Annotation System (Prokka) pipeline (v1.14.5)^3^ was uniformly applied to all the 121 *G. parasuis* genomes. Based on the GFF3 files produced by Prokka, the Roary program^4^ was used to construct the pan-genome with a minimum percentage identity of 95% between each predicted protein homolog.

### 2.3. Association of virulence with pan-genome

Serotypes were determined *via* PCR simulation amplification. *G. parasuis* serotypes PCR identified primers were used for PCR simulation amplification the genome of 121 *G. parasuis* to identify strains serotypes, as the basis for the virulence determination. Moreover, combining the strain background information recorded in the literature and NCBI, the 121 isolates were classified as either virulent and avirulent strains or virulent, moderate virulent and avirulent strains (Table 2). Furthermore, according to literature, serotype 7 strains previously considered nontoxic were classified in the virulence strain category. Subsequently, virulence traits were translated into binary (such as “1” is a virulent strain, and “0” is an avirulent strain) and inputted Scoary.^5^ Combined with the information on the presence or absence of genes in each isolate obtained by pan-genome analysis, the association between genes and virulence traits was judged based on Fisher’s exact test. *P* values determined significance between genes and traits. And Odds Radio (OR) greater than 1 as a criterion for judging the strength of the positive association. Moreover, the basis on pairwise comparison algorithms in the unweighted pairs group using mean average (UPGMA) trees, the number of genes and phenotypes in evolutionary history is taken as strong evidence for a causal association.

**Table 2:** Classification of the virulence of 121 *G. parasuis* strains.

Virulence classification	Serotype	Number of strains	Total
Virulent	1	6	67
	5	20	
	10	6	
	12	4	
	13	10	
	14	8	
	7	13	
Moderate virulent	2	10	35
	4	19	
	8	2	
	15	4	
Avirulent	3	1	6
	6	2	
	9	1	
	11	2	
Unknow	Unknow	13	13

The selected virulence trait association genes were used for Gene Ontology (GO) and Kyoto Encyclopedia of Genes and Genomes (KEGG) analysis, and the virulence factors were annotated. The final results were visualized using R 4.1.

### 2.4. Biofilm quantification with microtiter plate assay and pan-GWAS

The mediums used to culture bacteria were Trypticase Soy Agar (TSA, OXOID, Hampshire, England) media supplemented with 5% (v/v) calf serum and nicotinamide adenine dinucleotide (NAD, JS, Nanjing, China). Bacteria were routinely streaked from −80°C stocks onto TSA and incubated at 36 h at 37°C before each experiment. Then 20 μL of the bacterial cell suspension were inoculated in 2 mL of Trypticase Soy Broth (TSB, OXOID, Hampshire, England) medium containing 5% calf serum with NAD and added to 24-well plates. Sterile media were added as a negative control. Microplates were incubated statically for 48 h at 37°C. The media and planktonic cells were removed by gentle tapping *via* inverted microplates. To remove loosely attached bacteria, wells were washed twice with 300 μL of sterile PBS. Then biofilms were fixed with 500 μL of methanol for 30 min and air-dried completely at room temperature after removal of the methanol. In order to stain bacterial biofilm, 500 μL of 1% crystal violet (CV) solution was added per well and plates were incubated statically for 30 min at room temperature. Then the solution was removed after the bacterial biofilm was completely dyed. Wells were washed 3 times with 300 μL of saline and air-dried at 37°C before taking photos of the record. Wells were added 200 μL of 33% v/v acetic acid to dissolve CV in biofilm completely. The amount of destained CV was determined by reading OD_630_ in a microplate reader.

The basis on the comparison of OD_630_ value, 16 isolates were classified into strong biofilm-forming strains and weak biofilm-forming strains. Then, the biofilm forming ability of strains was translated into binary and inputted Scoary (see footnote 5). Combined with the information on the presence or absence of genes in each isolate obtained by pan-genome analysis, the association between genes and biofilm forming ability was judged based on Fisher’s exact test. The significance between genes and traits was determined by *p* values. And Odds Radio (OR) greater than 1 as a criterion for judging the strength of the positive association. Moreover, the basis of pairwise comparison algorithms in the unweighted pairs group using mean average (UPGMA) trees, the number of genes and phenotypes in evolutionary history is taken as strong evidence for a causal association.

The selected biofilm forming association genes were used for GO and KEGG enrichment. The final results were visualized using R 4.1.

### 2.5. Antimicrobial agent susceptibility testing

The minimum inhibitory concentrations (MICs) of 16 strains were tested as described previously studies (Zhang et al., 2019). Six drugs frequently were used to against *G. parasuis* were selected, namely, amoxicillin, ampicillin, gentamycin, kanamycin, and tetracycline. *Escherichia coli* ATCC 25922 was used as a quality control strain for drug susceptibility.

### 2.6. Cell adhesion experiments

Adhesion experiments were performed using PK-15 cells. The experiments were performed based on a previously described (Jiang C. et al., 2021) with some minor modifications. Briefly, the 5 × 10^5^ cells were seeded into 24-well plates in dulbecco’s modified eagle medium (DMEM, Gibco, California, United States) medium containing 10% fetal bovine serum (FBS, Gibco, California, United States) at 37°C in a humidified incubator containing 5% CO_2_. After the cells have grown to 100% confluence, the cells were washed thrice with PBS and infected with approximately 1 × 10^7^ CFU *G. parasuis*. The culture plates were thereafter incubated at 37°C for 2 h to allow bacterial adhesion. The plates were then washed five times with sterile PBS to eliminate non-specific bacterial attachment and then incubation with 200 μL 0.25% trypsin/EDTA at 37°C for 10 min. After incubation, the cells were resuspended from the bottom of every well. The cell suspensions with adherent bacteria were diluted by 10 times and placed onto the TSA (OXOID, Hampshire, United Kingdom) plates supplemented with NAD and serum and then incubation at 37°C for 12 h. And then cell counting was performed, and the adhesion rate was calculated.

## 3. Results

### 3.1. Separation and composition of the core genome and the accessory genome

In this study, the pan-genome was classified into the core genome and the accessory genome: 1,133 genes were included in the core genome (i.e., present in more than 95% of the strains, core and soft-core genes) and 7,752 genes in the accessory genome (i.e., less than 95% of the strains, shell and cloud genes). More detailed information is presented in Table 3.

**Table 3:** Characteristics of the core genome and the accessory genome.

Pan-genome	Classification	Number of genes	Max length (bp)	Min length (bp)	Average length (bp)
Core genome	Core genes	390	4,491	132	821.93
	Soft-core genes	743	4,029	123	952.79
Accessory genome	Shell genes	1880	17,223	123	890.51
	Cloud genes	5,872	11,295	123	715.25

Our results were consistent with previous studies that *G. parasuis* has an open pan-genome. The core genome of *G. parasuis* is small, accounting for only 4.39% of the pan-genome. The rest of the genome is highly variable, continuously obtaining foreign genes to adapt to different environments.

### 3.2. The COG functional classification of the core genome and the accessory genome

**Figure 1:**
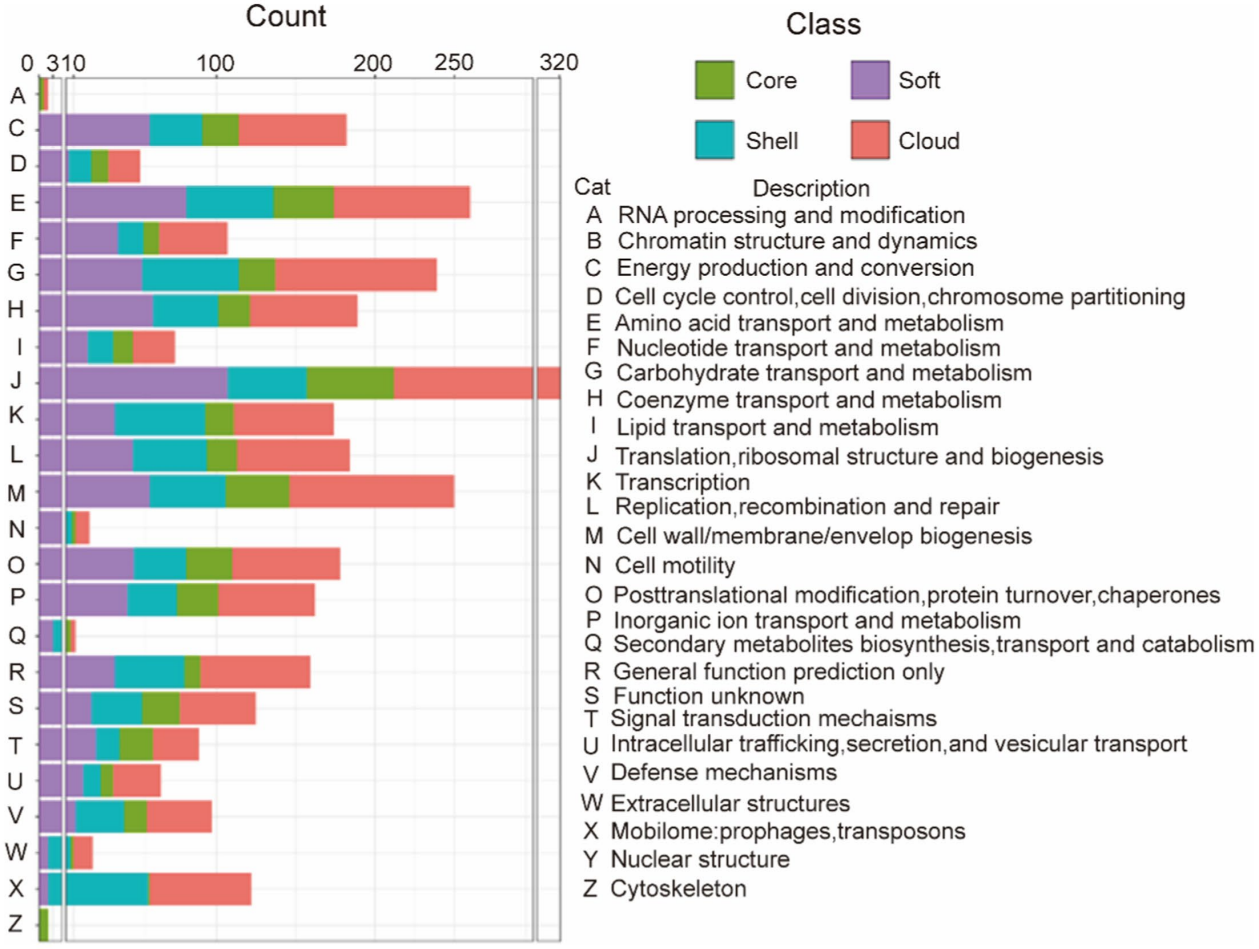
COG annotation of the core genome and the accessory genome. The category is shown in Table 2, green represents the core gene, purple represents the soft-core gene, blue represents the shell gene, and red represents cloud genes.

In order to study the functions of the different gene sets, we used an eggNOG-mapper to align and annotate with clusters of orthologous groups (COG). By clustering the analysis of the COG function, we found that the backbone of the genome and the basic biological phenotypes consisted of the core genome (Table 4 and Figure 1). The core genome participates in all link of the bacterial life process and all function classification includes some proportion of the core genome. The primary function of the core genome focuses on the E (Amino-acid transport and metabolism), J (Translation, ribosomal structure, and biogenesis), and T (Signal transduction mechanisms) of COG functional classifications. Particularly, the cytoskeleton was only found in the core genes, suggesting that the core genes play an important role in maintaining the cell morphology and the internal cell structure. The Accessory genome includes all COG functional classifications except cytoskeleton (Table 4 and Figure 1). It means that the accessory genome will also participate in a part of the life process. Moreover, the major function of the accessory genome focuses on the X (Mobilome: prophages, transposons), V (Defense mechanisms), and G (Carbohydrate transport and metabolism) of COG functional classifications. These functions may confer selective advantages to *G. parasuis* and enrich population diversity (Supplementary Table S3). Notably, up to ~17.11% of the core and accessory genome still have unknown functions in the COG database (Table 4).

**Table 4:** COG annotation of the core genome and the accessory genome.

Classifications	Functions	Core	Soft_core	Shell	Cloud
A	RNA processing and modification	1 (0.24%)	NA	NA	1 (0.08%)
C	Energy production and conversion	23 (5.58%)	58 (7.47%)	33 (4.7%)	68 (5.69%)
D	Cell-cycle control, cell division, and chromosome assignment	11 (2.67%)	7 (0.9%)	14 (1.99%)	20 (1.67%)
E	Amino acid transport and metabolism	38 (9.22%)	81 (10.44%)	55 (7.83%)	86 (7.19%)
F	Nucleotide transport and metabolism	10 (2.43%)	38 (4.9%)	16 (2.28%)	43 (3.6%)
G	Carbohydrate transport and metabolism	23 (5.58%)	53 (6.83%)	61 (8.69%)	102 (8.53%)
H	Coenzyme transport and metabolism	20 (4.85%)	60 (7.73%)	41 (5.84%)	68 (5.69%)
I	Lipid transport and metabolism	12 (2.91%)	19 (2.45%)	16 (2.28%)	27 (2.26%)
J	Translation, ribosomal structure and biogenesis	55 (13.35%)	107 (13.79%)	50 (7.12%)	109 (9.11%)
K	Transcription	18 (4.37%)	36 (4.64%)	57 (8.12%)	63 (5.27%)
L	Replication, recombination and repair	19 (4.61%)	47 (6.06%)	47 (6.7%)	71 (5.94%)
M	Cell wall/membrane/envelope biogenesis	40 (9.71%)	58 (7.47%)	48 (6.84%)	104 (8.7%)
N	Cell motility	2 (0.49%)	5 (0.64%)	4 (0.57%)	9 (0.75%)
O	Posttranslational modification, protein turnover, chaperones	29 (7.04%)	48 (6.19%)	33 (4.7%)	68 (5.69%)
P	Inorganic ion transport and metabolism	26 (6.31%)	44 (5.67%)	31 (4.42%)	61 (5.1%)
Q	Secondary metabolites biosynthesis, transport and catabolism	3 (0.73%)	3 (0.39%)	2 (0.28%)	3 (0.25%)
R	General function prediction only	10 (2.43%)	36 (4.64%)	44 (6.27%)	69 (5.77%)
S	Function unknown	24 (5.83%)	21 (2.71%)	32 (4.56%)	48 (4.01%)
T	Signal transduction mechanisms	21 (5.1%)	24 (3.09%)	15 (2.14%)	29 (2.42%)
U	Intracellular trafficking, secretion, and vesicular transport	8 (1.94%)	16 (2.06%)	11 (1.57%)	30 (2.51%)
V	Defense mechanisms	14 (3.4%)	11 (1.42%)	31 (4.42%)	41 (3.43%)
W	Extracellular structures	2 (0.49%)	2 (0.26%)	6 (0.85%)	12 (1%)
X	Mobilome: prophages, transposons	1 (0.24%)	2 (0.26%)	55 (7.83%)	64 (5.35%)
Z	Cytoskeleton	2 (0.49%)	NA	NA	NA

### 3.3. The PHI annotation of the core genome and the accessory genome

**Figure 2:**
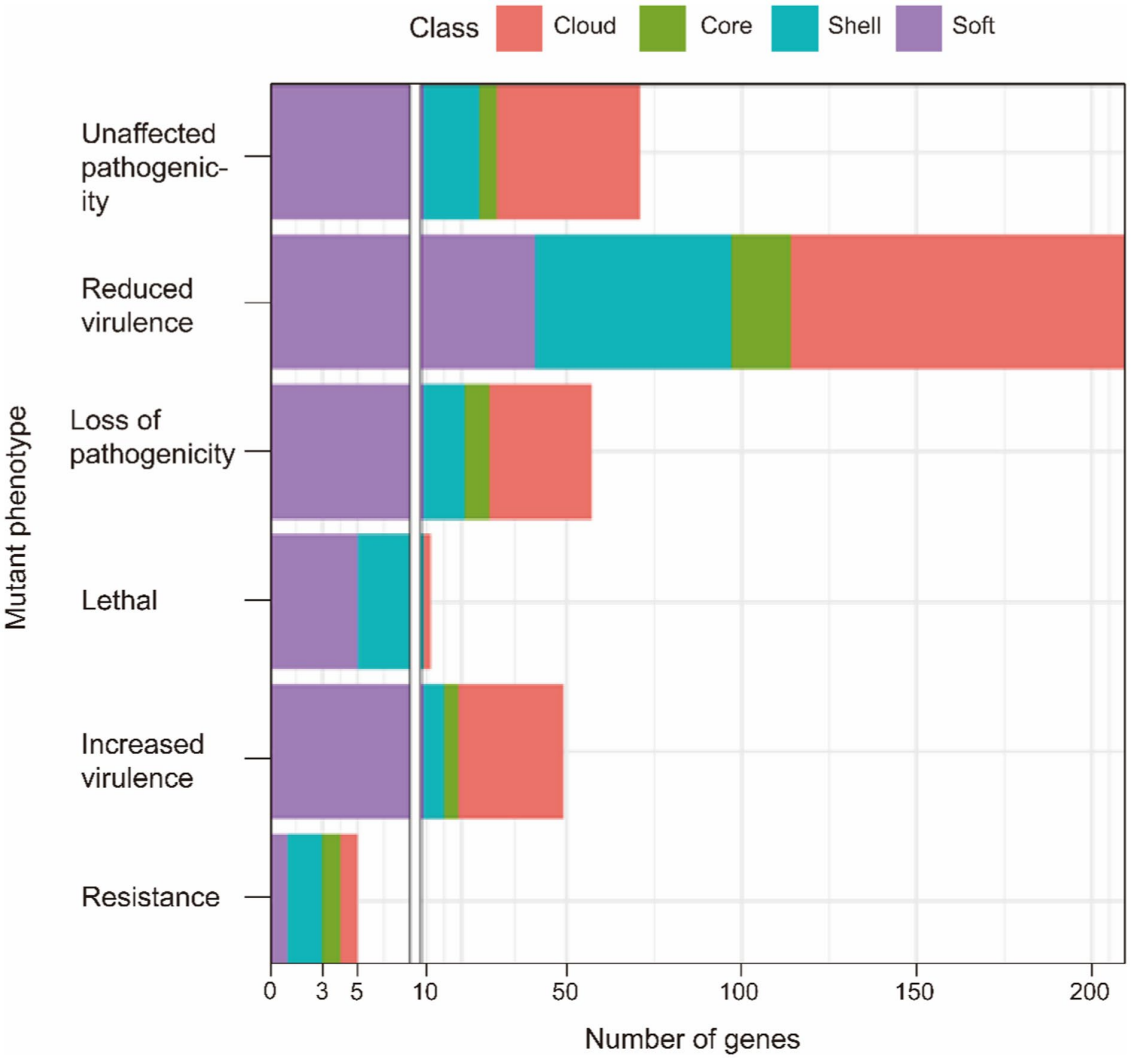
PHI annotation of the core genome and the accessory genome.

Pathogen-host interactions are usually the basis of the occurrence of infectious diseases. In this study, BLASTp was used to confirm genes related to pathogen-host interactions (PHI). Four classes of gene sets were aligned to the pathogen-host interaction database and annotated. Our results showed that although pathogenic genes are widely found in both core and accessory genome, the accessory genome has a higher abundance than the core genome. All related genes annotated to Glasser’s disease are mainly present in the accessory genome. These genes relate to bacteria’s serum resistance, adhesion, and invasion capabilities. Additionally, lethal factors were not found in the core gene. This result also provided evidence that the primary function of the core genes is to perform the necessary biological processes (as shown in Figure 2 and Table 5). And more detailed information are summarized in Supplementary Table S4.

**Table 5:** PHI annotation of the core genome and the accessory genome.

Mutant phenotype	Core	Soft	Shell	Cloud
Resistance	1	1	2	1
Increased virulence	4	9	6	30
Loss of pathogenicity	7	9	12	29
Reduced virulence	17	41	56	96
Unaffected pathogenicity	5	9	16	41
Lethal	0	5	4	2

### 3.4. The VFDB annotation of the core genome and the accessory genome

**Figure 3:**
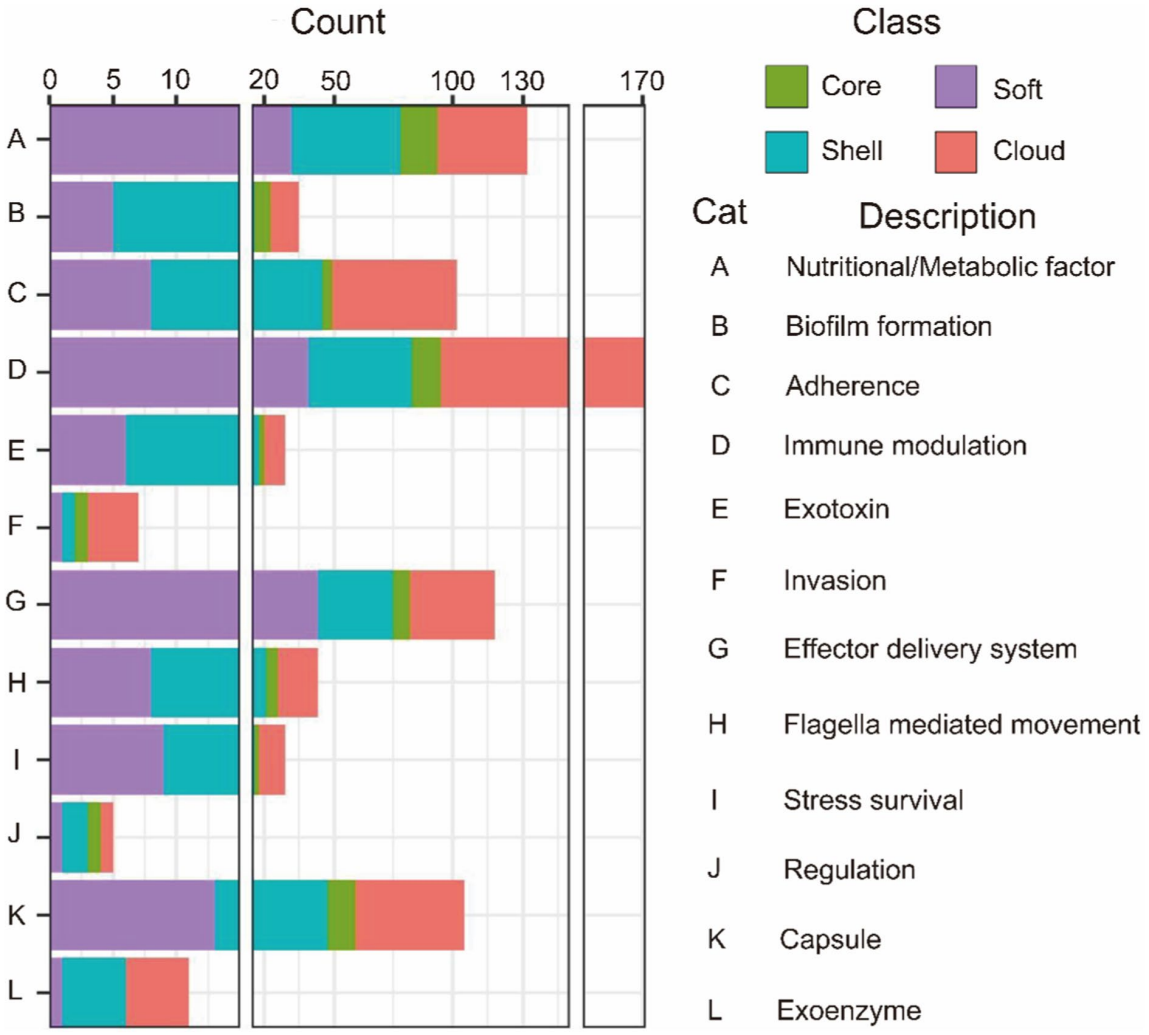
VFDB annotation of the core genome and the accessory genome.

The virulence genes of pathogenic bacteria encode toxins, adhesins or other virulence factors. Four gene sets were compared with Virulence factors of pathogenic bacteria (VFDB) databases and annotated to identify potential virulence factors. In VFDB-annotation genes, the core genome was found to exist in all classifications. Interestingly, compared with the accessory genome, the core genome has the highest proportion of nutritional/metabolic factors, biofilm formation, and effector delivery system (Table 6). These cases supported our previous conclusion. Namely, the functions of the core genome focus on maintaining survival. Additionally, the core genome is essential for bacteria to interfere with host defenses or regulate their invasion (Figure 3).

**Table 6:** VFDB annotation of the core genome and the accessory genome.

Classfications	Core	Soft	Shell	Cloud
Nutritional/Metabolic factor	16	32	46	38
	(23%)	(19%)	(19%)	(16%)
Biofilm formation	7	5	11	12
	(10%)	(3%)	(5%)	(5%)
Adherence	4	8	37	53
	(6%)	(5%)	(15%)	(22%)
Immune modulation	12	39	44	76
	(17%)	(23%)	(18%)	(31%)
Exotoxin	2	6	12	9
	(3%)	(4%)	(5%)	(4%)
Invasion	1	1	1	4
	(1%)	(1%)	(1%)	(2%)
Effector delivery system	7	43	32	36
	(10%)	(26%)	(13%)	(15%)
Flagella mediated movement	5	8	13	17
	(7%)	(5%)	(5%)	(7%)
Stress survival	2	9	7	11
	(3%)	(5%)	(3%)	(5%)
Regulation	1	1	2	1
	(1%)	(1%)	(1%)	(1%)
Capsule	12	13	34	46
	(17%)	(8%)	(14%)	(19%)
Exoenzyme	NA	1	5	5
		(1%)	(2%)	(2%)

In contrast, virulence factors of the accessory genome mainly focus on B (Biofilm formation), C (Adherence), D (Immune modulation), F (Invasion), and K (Capsule). The core and accessory genomes showed differences in the composition of adherence-associated genes. For example, bartonella adhesin A (BadA)/ variably expressed outer-membrane proteins (Vomp), belonging to cloud genes, can promote bacterial autoaggregation as well as the adhesion of extracellular matrix proteins, resulting in activating hypoxia-inducible factor-1 (HIF-1) and inducing the secretion of vascular endothelial growth factor (VEGF) secretion in infected host cells (Riess et al., 2004; Zhang et al., 2004; Kaiser et al., 2008). Additionally, BadA/Vomp also is necessary to format optimal biofilm (Okaro et al., 2019). Interestingly, BadA/Vomp is not included in the core genome, but the reason for the difference is unknown.

Differences in immune modulation and stress survival between the core and accessory genome included virulence factors such as neisserial surface protein A (NspA), RecN. NspA play an essential role in binding complement factor H to inhibit host innate immune defenses (Lewis et al., 2010, 2012). RecN, a recombinational repair protein, protects against ROS and non-oxidative killing by neutrophils (Stohl et al., 2005; Criss et al., 2009). These virulence factors are beneficial for bacterial survival in the host to trigger subsequent infection.

Virulence factors for exoenzyme were also found. IgA1 protease can interfere with the barrier functions of mucosal IgA antibodies *via* cleaving secretory IgA1 in the hinge region (Rao et al., 1999). Hyaluronidase, an essential pathogenic bacterial spreading factor, can cleave hyaluronan to help bacterial spread (Baker and Pritchard, 2000; Li and Jedrzejas, 2001). Interestingly, these virulence factors classed into exoenzyme were only found in the accessory genome. Our results could indicate exoenzyme produced by the accessory genome is the dominant extracellular digestive function. This process is beneficial for the survival and spread of bacteria in the host.

Thus, various types of virulence factors were found in the core gene. The avirulent strains considered in earlier studies may also contribute to the infection process. Moreover, some avirulent strains may transfer into virulent strains *via* obtaining foreign genetic material. Virulence factors in the accessory genome are complement to the core genome. These virulence factors confer *G. parasuis* various pathogenic mechanisms to provide the opportunity for bacterial survival, resulting in more severe pathological responses in the host (Figure 3 and Table 6).

### 3.5. The CARD annotation of the core genome and the accessory genome

**Figure 4:**
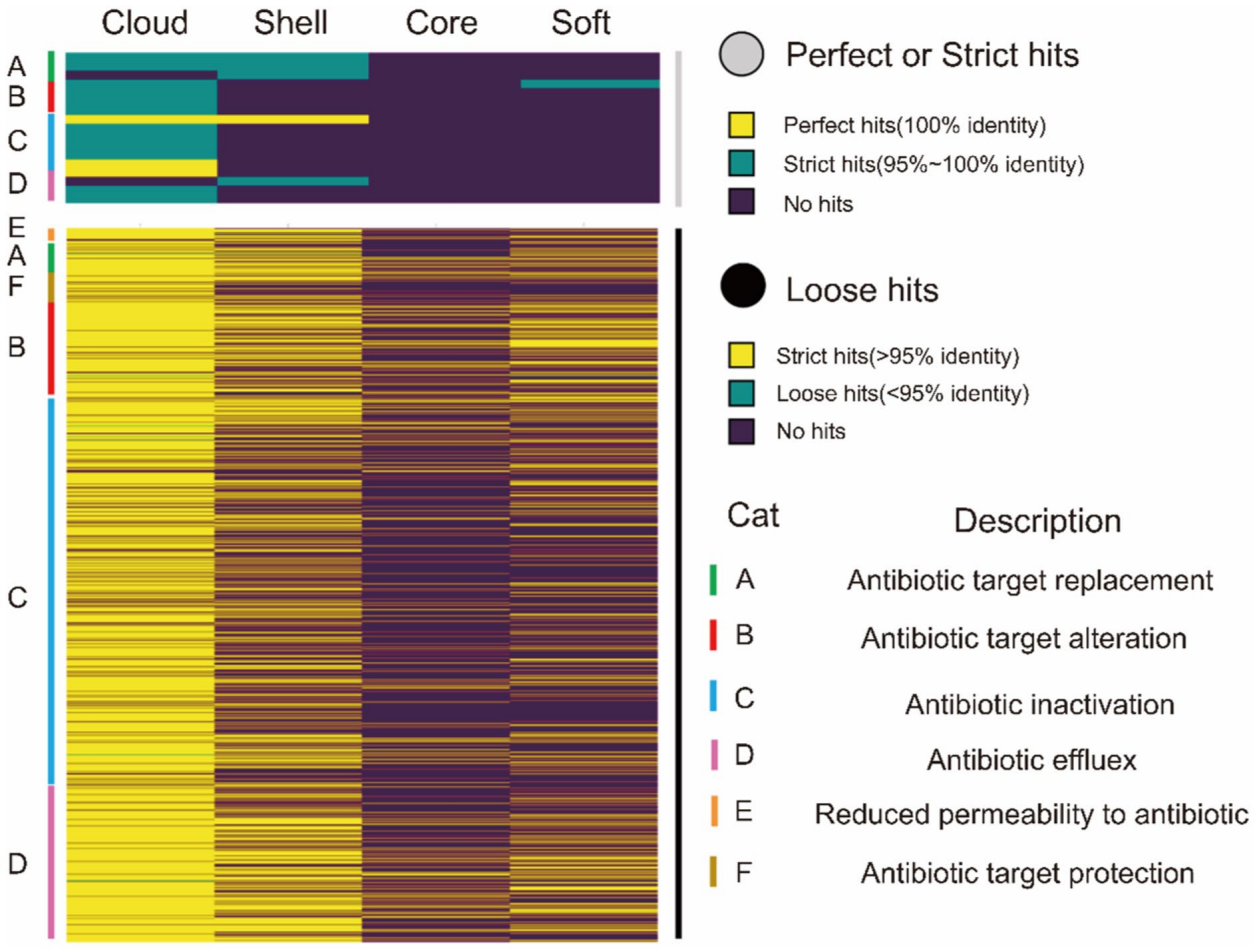
CARD annotation of the core genome and the accessory genome. The upper heatmap (gray) is the annotation result obtained using perfect or strict mode, yellow represents 100% agreement, green represents 95 ~ 100% agreement, and purple represents no match. The lower heatmap shows the annotation results obtained using loose mode, with >95% agreement in yellow, <95% agreement in green, and no match in purple. The right side of the heat map is marked as A ~ F, and there are 6 types of drug resistance mechanisms.

In relation to the resistance genes, the resistance gene identifier (RGI) was used to analyze four classes of gene sets. Figure 4 shows the proportion of different resistance mechanisms in perfect or strict hits or loose hits for the four gene sets.

The resistance genes were completely missing in the core genes in perfect or strict hits. Similarly, we only identified one gene about cephalosporin in the soft-core gene of the core genome. However, the accessory genome abundantly enriched other resistance genes (diaminopyrimidine, cephalosporin, peptide, sulfonamide, tetracycline, and lincosamide). Moreover, these resistance genes characteristic of the accessory genome was classified into four resistance mechanisms: A (antibiotic target replacement), B (antibiotic target alteration), C (antibiotic inactivation), and D (antibiotic efflux) (Table 7).

**Table 7:** CARD annotation of the core genome and the accessory genome (Perfect or Strict hits).

Class	Gene	Drug class	Resistance mechanism	AMR gene family
Core	NA	NA	NA	NA
Soft-core	*ftsI*	Cephalosporin	Antibiotic target alteration	Penicillin-binding protein mutations
Shell	*dhfrIII*	Diaminopyrimidine	Antibiotic target replacement	Trimethoprim resistant dihydrofolate reductase dfr
	*GPS_2460*	Diaminopyrimidine	Antibiotic target replacement	Trimethoprim resistant dihydrofolate reductase dfr
	*penP*	Cephalosporin	Antibiotic inactivation	ROB beta-lactamase
	*folP_2*	Sulfonamide	Antibiotic target replacement	Sulfonamide resistant dihydropteroate synthase folP
	*tetR*	Tetracycline	Antibiotic target alteration	Major facilitator superfamily (MFS) antibiotic efflux pump
			Antibiotic efflux	
Cloud	*GPS_8640*	Cephalosporin	Antibiotic target alteration	Penicillin-binding protein mutations
	*GPS_8664*	Cephalosporin	Antibiotic target alteration	Penicillin-binding protein mutations
	*GPS_2847*	Peptide	Antibiotic target alteration	MCR phosphoethanolamine transferase
	*tetA*	Tetracycline	Antibiotic efflux	Major facilitator superfamily (MFS) antibiotic efflux pump
	*GPS_4453*	Tetracycline	Antibiotic efflux	Major facilitator superfamily (MFS) antibiotic efflux pump
	*GPS_4456*	Tetracycline	Antibiotic efflux	Major facilitator superfamily (MFS) antibiotic efflux pump
	*GPS_4455*	Tetracycline	Antibiotic efflux	Major facilitator superfamily (MFS) antibiotic efflux pump
	*linA*	Lincosamide	Antibiotic inactivation	Lincosamide nucleotidyltransferase (LNU)
	*folP_1*	Sulfonamide	Antibiotic target replacement	Sulfonamide resistant dihydropteroate synthase folP
	*GPS_5385*	Sulfonamide	Antibiotic target replacement	Sulfonamide resistant dihydropteroate synthase folP
	*GPS_5386*	Sulfonamide	Antibiotic target replacement	Sulfonamide resistant dihydropteroate synthase folP
	*cat*	Phenicol	Antibiotic inactivation	Chloramphenicol acetyltransferase (CAT)
	*bcr_2*	Phenicol	Antibiotic efflux	Major facilitator superfamily (MFS) antibiotic efflux pump
	*GPS_6310*	Diaminopyrimidine	Antibiotic target replacement	Trimethoprim resistant dihydrofolate reductase dfr
	*dhfrIII_2*	Diaminopyrimidine	Antibiotic target replacement	Trimethoprim resistant dihydrofolate reductase dfr
	*GPS_8666*	Diaminopyrimidine	Antibiotic target replacement	ROB beta-lactamase
	*GPS_8892*	Diaminopyrimidine	Antibiotic target replacement	ROB beta-lactamase
	*GPS_7895*	Aminoglycoside	Antibiotic inactivation	APH(6)
	*neo*	Aminoglycoside	Antibiotic inactivation	APH(3″)
	*aacA-aphD*	Aminoglycoside	Antibiotic inactivation	APH(2″); AAC(6″)
	*GPS_7899*	Aminoglycoside	Antibiotic inactivation	APH(3″)
	*GPS_7898*	Aminoglycoside	Antibiotic inactivation	APH(3″)
	*GPS_7894*	Aminoglycoside	Antibiotic inactivation	APH(6)
	*GPS_7901*	Aminoglycoside	Antibiotic inactivation	APH(3″)
	*ant1*	Aminoglycoside	Antibiotic inactivation	ANT(3″)
	*yokD*	Aminoglycoside	Antibiotic inactivation	AAC(3)
	*GPS_6438*	Aminoglycoside	Antibiotic inactivation	APH(3″)
	*GPS_6441*	Aminoglycoside	Antibiotic inactivation	APH(3″)
	*GPS_7896*	Aminoglycoside	Antibiotic inactivation	APH(6)
	*GPS_7897*	Aminoglycoside	Antibiotic inactivation	APH(6)
	*ftsI_2*	Cephalosporin	Antibiotic target alteration	Penicillin-binding protein mutations
	*GPS_8575*	Cephalosporin	Antibiotic target alteration	Penicillin-binding protein mutations
	*GPS_8640*	Cephalosporin	Antibiotic target alteration	Penicillin-binding protein mutations
	*GPS_8664*	Cephalosporin	Antibiotic target alteration	Penicillin-binding protein mutations

NA stands for missing values.

In loose hits, although resistance genes involved in six resistance mechanisms were found in both core and soft-core gene, the proportion of resistance genes is still lower than in the accessory genome (Figure 4). As in the results in perfect or strict hits, the resistance genes are mainly concentrated in the accessory genome. Our results showed that resistance genes of *G. parasuis* mostly come from the accessory genome. The genome is a dynamic repository of genetic information. The constant gain and loss of resistance genes are beneficial for bacteria to adapt to environmental stress and maintain survival in the host. Furthermore, the dynamic resistance profile also makes the clinical treatment of *G. parasuis* difficult.

### 3.6. The GO annotation and enrichment of the core genome and the accessory genome

BLASTx was used to compare all genes in the pan-genome with the Swiss-Prot database. The GO annotation of all genes was completed by comparing the above results with bacterial GO mapping background information. According to GO annotation files, an enricher was called to achieve the enrichment analysis of the four gene sets. *P* value (<0.05) was used to judge the significance.

**Figure 5:**
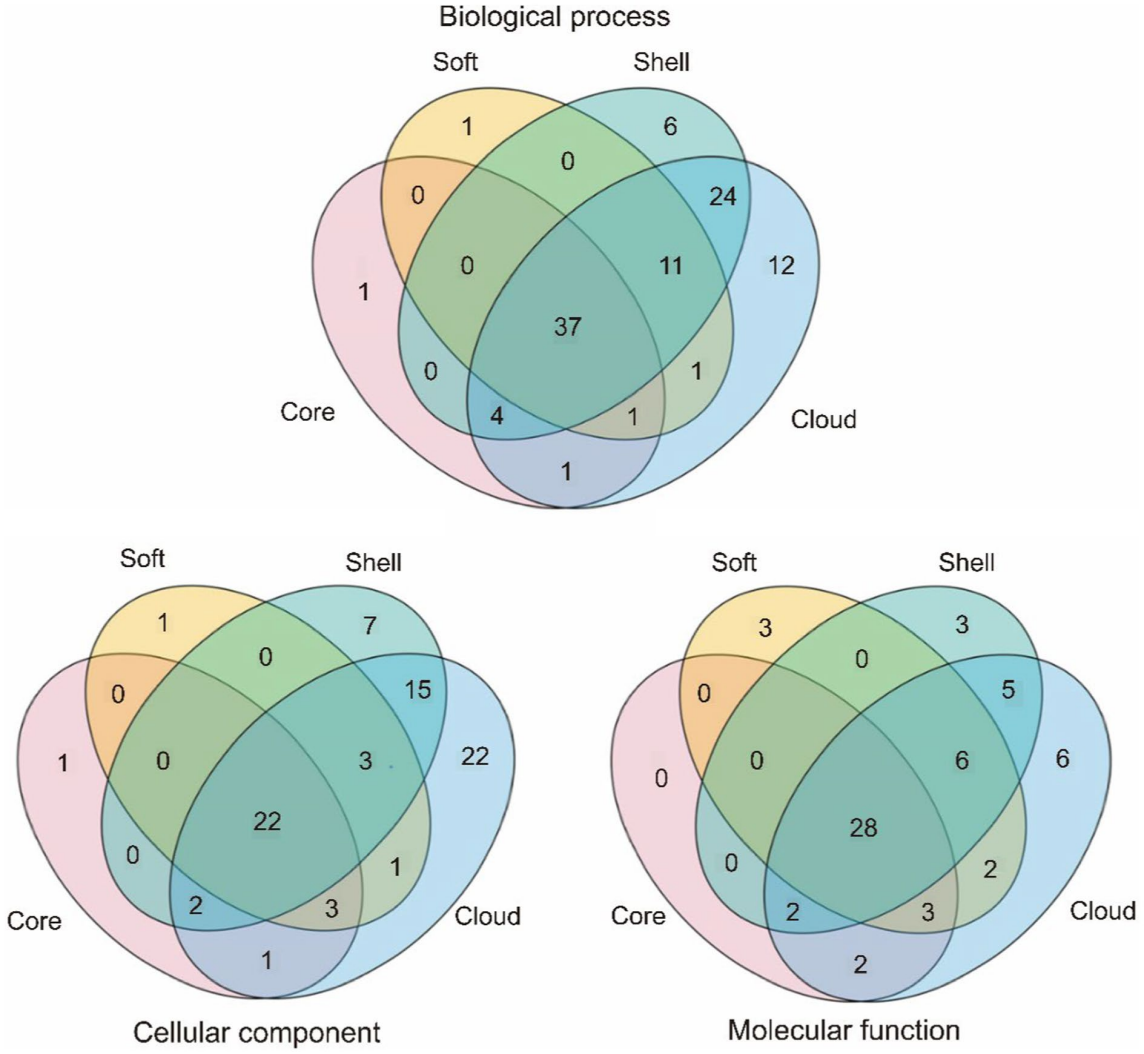
Comparison of GO annotation of the core genome and the accessory genome.

Our results showed that the major structures of bacteria consist of the core genome (Supplementary Figure S1). These structures include cytoderm, cytomembrane, membrane protein complex, ribosome, etc. The core genome also participates in various biological processes of bacterial survival, including DNA replication, transcription, and translation. Moreover, the synthesis and metabolism of various necessary substances are involved in the core genome, such as RNA, amino acid, lipid, organic acid, etc. Overall, the core genome is an essential part of the basic structure and life activities of *G. parasuis* (Figure 5).

Compared to the core genome, the majority of genes characteristic of the accessory genome focus on the host cells binding, extracellular matrix binding, quinone binding, proteasome activity and so on. In addition, the core genome and the accessory genome also showed differences in biological processes. Compared to the core genome, the accessory genome pays attention to dispensable biological processes including colonization, parasitism, defense reaction, anaerobic respiration and so on. This study showed that bacterial and host interrelation of abilities are given by the accessory genome. Furthermore, genes for defense against phage infection were also found in the accessory genome. The accessory genome renders bacteria to adapt the metabolic way to survive in different environments.

### 3.7. The KEGG annotation and enrichment of the core genome and the accessory genome

Kyoto Encyclopedia of Genes and Genomes (KEGG) annotation and enrichment is an effective method to search for the correlation between genes and signaling pathways. In this study, clusterProfiler was used for KEGG enrichment of the core and the accessory genome.

**Figure 6:**
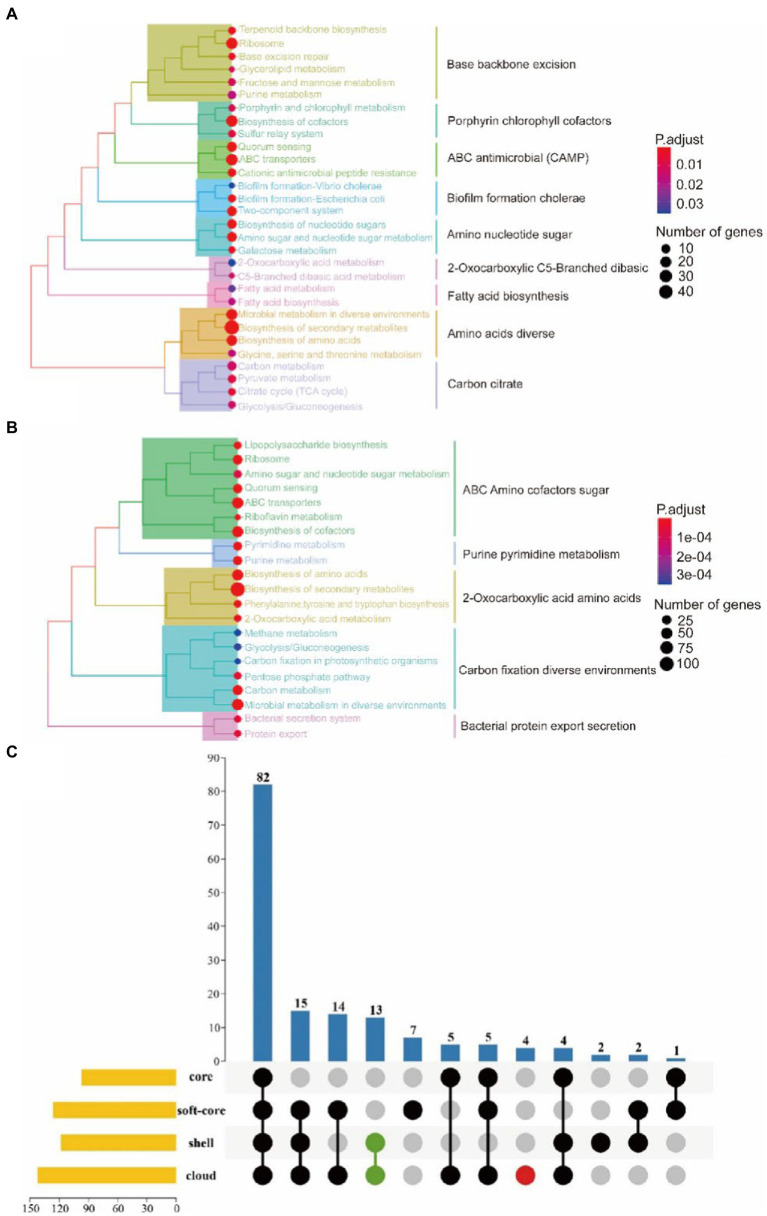
KEGG enrichment of the core genome. **(A)** Represents the core gene. **(B)** Represents the soft-core gene. **(C)** Represents KEGG enrichment for the four classes of gene sets. The yellow bar chart on the left represented the number of KEGG metabolic pathways involved in each of the four gene sets. The bar chart at the top and the dot chart at the bottom together explained the number of KEGG metabolic pathways involved in different groups of the four gene sets. Additionally, green and red were used to represent the accessory genomes and cloud genes, respectively.

The core genome is mainly involved in metabolism, genetic information processing, environmental information processing, cellular processes, and other signaling pathways (Figures 6A,B). Notably, the quorum sensing system and biofilm formation are one of the functional pathways involved in the core genome of *G. parasuis*. It showed that all *G. parasuis* strains had the ability to form biofilms. This ability helps bacteria resist stress and maximize energy to ensure colony survival. Furthermore, the core genome also showed resistance to cationic antimicrobial peptides and vancomycin. These resistances are beneficial for the bacteria to overcome the innate immune system in the host engraftment.

In relation to the accessory genome, this study also found specific signal pathways, including ethylbenzene degradation, naphthalene degradation, chlorinated dilute hydrocarbon degradation, arabinogalactan biosynthesis, and sphingolipid metabolism (Figure 6C). Functional genes related-pathways may be obtained from the environment by bacteria. The abundant carbon source utilization pathway facilitates the survival of *H.parasuis* in the low-oxygen lung environment or inside the biofilm in pathological conditions, resulting in long-term infection. Arabinogalactan participates in cell wall assembly to provide an antibiotic penetration barrier to bacterial (Degnan and Macfarlane, 1995). In addition, sphingolipid metabolism is beneficial for *G. parasuis* to interrupt host’s sphingolipid balance leading to colonization, invasion, and intracellular survival of bacterial.

### 3.8. Virulence and pan-genome-wide association

**Figure 7:**
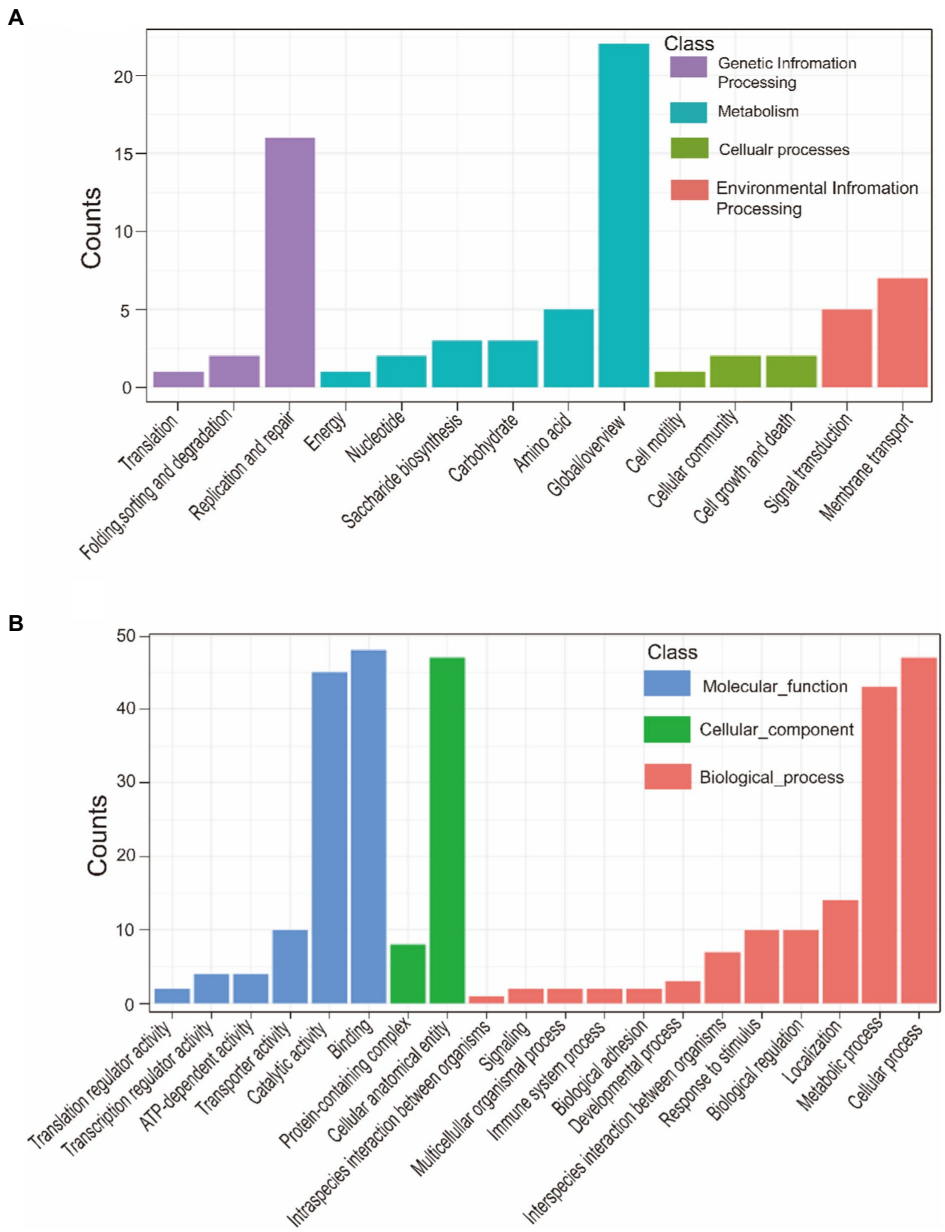
KEGG annotation of strong virulence-associated genes **(A)**. GO annotation of strong virulence-associated genes **(B)**.

A total of 121 strains were classified into virulent strains and avirulent strains. Virulence traits were grouped into binary categories for Pan-GWAS. We resulted that constant virulence factor was not found in virulence strains. This result supports that the bacterial genome is a viewpoint of a dynamic gene repository. A total of 142 genes were found to be associated with strong virulence traits in this study, involved in signal pathways such as metabolism, genetic information processing, environmental information processing, and cellular processes (Supplementary Table S5 and Figure 7). Metabolic pathways support the maintenance of normal bacterial life activities, including carbohydrate metabolism, energy metabolism, amino acid metabolism, etc. Differences in the functional pathways of genetic information processing include DNA repair, mismatch repair, homologous recombination, etc. These functional pathways showed that exogenous genetic material might be easily obtained by virulent strains. Moreover, virulent strains may have stronger adaptability to harsh environment to decrease survival pressure. Differences in the functional pathways of environmental information processing include phosphotransferase (PTS) systems and the two-component systems (TCS), which can be used for signal transduction and sensing of environmental stimuli. Other pathways such as the secretory system, quorum sensing system, and ABC transporter system take effect in bacterial colonization and acquisition of niche competitive advantage.

This study also showed the main functional categories of the virulence factors (Table 8). (1) Adherence is helpful for virulent strains colonization in the host. (2) Nutritional/metabolic, essential nutrients are necessary to colonize the host successfully. For instance, bacteria can compete or usurp iron ion from the host by utilizing heme, transferrin, and lactoferrin (Faraldo-Gómez and Sansom, 2003). (3) Exotoxin is beneficial to resist the clearance of the host immune system *via* inducing host cell apoptosis and cleaving of macrophages. (4) Regulation. For example, bacteria can regulate their growth and metabolism in response to oxygen starvation. (5) Invasion. (6) Immune modulation. It is mediated by the capsule, lipo-oligosaccharide (LOS), lipopolysaccharide (LPS) and so on. The capsule has anti-phagocytosis and anti-complement effects of maintaining bacterial long-term survival in the host (Schembri et al., 2004). LOS participates in immune evasion and is a major proinflammatory factor. LOS is helpful for bacterial survival in different microenvironments within the host (Preston et al., 1996). (7) Secretory system can lead bacterial effector proteins to enter the host cells to manipulate their cellular processes.

**Table 8:** VFDB annotation of strong virulence-associated genes.

Gene	Production	Function
*upaG_10*	Autotransporter adhesin	Adherence
*group_8590*	SiM protein	Adherence
*elfC*	F17 fimbrial uscher	Adherence
*metR*	Transcriptional regulator	Nutritional/Metabolic-iron acquisition
*fhuB*	Fe (3+)-hydroxamate ABC transporter permease	Nutritional/Metabolic-iron acquisition
*hemY*	Heme biosynthesis protein	Nutritional/Metabolic-iron acquisition
*hxuC*	Heme-hemopexin utilization protein	Nutritional/Metabolic-iron acquisition
*group_3905*	Transferrin-binding protein 2 precursor	Nutritional/Metabolic-iron acquisition
*uhpT*	Hexose phosphate transport protein	Nutritional/Metabolic-carbohydrate
*uhpC*	Hexose phosphate transport protein	Nutritional/Metabolic-glucose
*glnA*	Type I glutamate-ammonia ligase	Nutritional/Metabolic
*oppA_1*	Peptide ABC transporter substrate	Nutritional/Metabolic
*lacE*	ABC transporter substrate	Nutritional/Metabolic
*group_5852*	Hemolysin A	Exotoxin
*group_2488*	RTX toxin transporter, ATPase protein	Exotoxin
*uup*	ABC-type transporter	Exotoxin
*prpC*	Serine–threonine phosphatase	Regulation
*uhpA*	Response regulator transcription factor	Regulation-oxidative stress
*group_6939*	HigA family addiction module antidote protein	Regulation
*group_1744*	Invasion protein IbeA	Invasion
*group_5547*	Membrane protein	Immune modulation -capsule
*group_7550*	Sialyltransferase	Immune modulation-LOS
*pglH*	Glycosyltransferase family 4 protein	Immune modulation -capsule
*group_4165*	Hypothetical protein	Immune modulation -capsule
*cpsY*	Capsular polysaccharide phosphotransferase	Immune modulation -capsule
*group_6341*	Glycosyltransferase	Immune modulation -capsule
*wbbD_1*	Glycosyltransferase	Immune modulation -capsule
*group_1181*	Polysaccharide biosynthesis protein	Immune modulation -capsule
*group_4155*	Glycosyltransferase family 4 protein	Immune modulation-LPS
*group_576*	Pyridoxal phosphate	Immune modulation-LPS
*arnB*	Lipopolysaccharide biosynthesis protein	Immune modulation-LPS
*epsL*	Probable sugar transferase	Immune modulation-LPS
*lpxK*	Tetraacyldisaccharide 4′-kinase	Immune modulation-LOS
*group_4170*	Sialyltransferase	Immune modulation-LOS
*lst_2*	Sialyltransferase	Immune modulation-LOS
*group_158*	Hyperosmolarity resistance protein	Immune modulation
*rbsB_2*	Sugar ABC transporter substrate	Secrete system
*tsf*	Type IV secretion system effector	Secrete system

### 3.9. Biofilm formation ability and pan-genome-wide association

**Figure 8:**
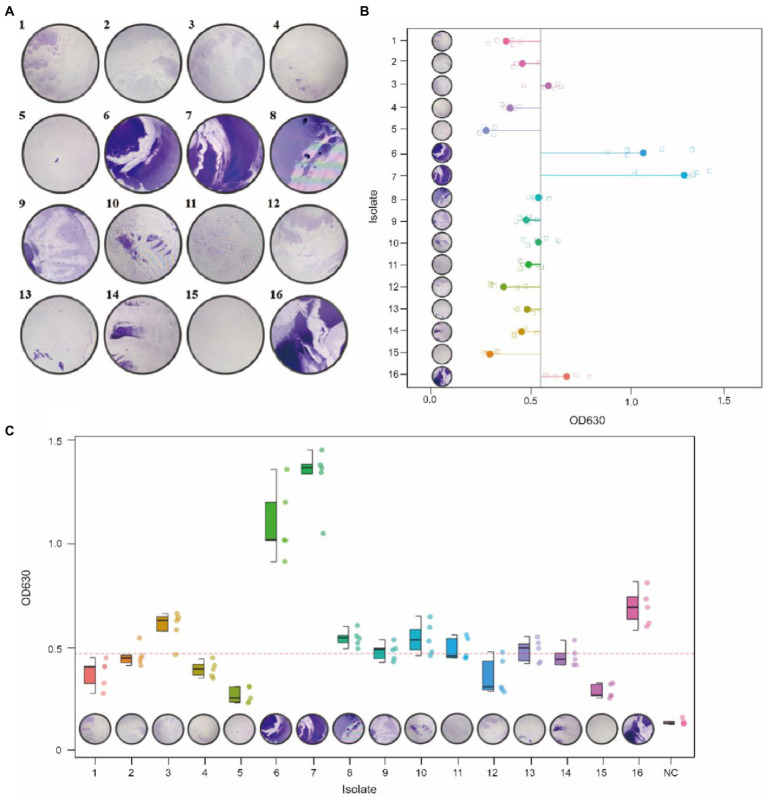
Crystal violet staining of biofilms of 16 *G. parasuis* strains **(A)**. One represents strain GP 01, others are analogous; the picture was taken from the biofilm formed on a 9 cm × 9 cm polystyrene petri dish. Comparison of biofilm forming ability of 16 *G. parasuis* strains **(B,C)**. **(B,C)** Described the minimum, 25th percentile, median, 75th percentile, and maximum values of OD 630 for 16 strains using scatter plots and boxplots, respectively. And jitters were added to prevent data overlap.

The biofilm formed by 16 *G. parasuis* strains on polystyrene plates is shown in Figure 8A. The OD_630_ values of biofilms of each strain were compared with those of the control group (TSB medium) by one-tailed hypothesis tests, and the *p* values were all lower than the test level (*α* = 0.05). Our results showed that all strains could form biofilm, which also supports previous conclusions on KEGG enrichment (Figure 8 and Table 9). According to the OD630 threshold of 0.5, 16 *G. parasuis* strains were divided into strong biofilm forming strains and weak biofilm forming strains. The results were classified into the binary category for Pan-GWAS (Figures 8B,C).

**Table 9:** Comparison of biofilm forming ability of 16 *G. parasuis* strains.

Strains	OD630	Mean ± Deviation var	*p*-value	Strains	OD630	Mean ± Deviation var	*p*-value
GP 01	0.466	0.3904 ± 0.069	3.92512E-05	GP 09	0.511	0.4944 ± 0.043	8.40868E-08
	0.343				0.443		
	0.296				0.504		
	0.425				0.552		
	0.422				0.462		
GP 02	0.478	0.475 ± 0.052	5.53427E-07	GP 10	0.503	0.5588 ± 0.043	1.32444E-06
	0.442				0.6		
	0.562				0.551		
	0.465				0.664		
	0.428				0.476		
GP 03	0.661	0.6112 ± 0.078	6.82489E-07	GP 11	0.557	0.5072 ± 0.054	3.14836E-07
	0.592				0.464		
	0.483				0.466		
	0.644				0.475		
	0.676				0.574		
GP 04	0.434	0.4114 ± 0.037	2.70049E-07	GP 12	0.306	0.3776 ± 0.087	0.000266419
	0.46				0.313		
	0.383				0.327		
	0.369				0.493		
	0.411				0.449		
GP 05	0.274	0.2866 ± 0.038	5.47184E-05	GP 13	0.451	0.4998 ± 0.054	4.07263E-07
	0.254				0.531		
	0.25				0.567		
	0.329				0.512		
	0.326				0.438		
GP 06	1.361	1.1066 ± 0.175	1.01956E-06	GP 14	0.431	0.4714 ± 0.049	3.77525E-07
	0.921				0.549		
	1.025				0.432		
	1.204				0.487		
	1.022				0.458		
GP 07	1.061	1.3214 ± 0.151	6.947E-08	GP 15	0.345	0.3054 ± 0.033	8.50377E-06
	1.339				0.338		
	1.385				0.273		
	1.369				0.287		
	1.453				0.284		
GP 08	0.561	0.5586 ± 0.04	1.19678E-08	GP 16	0.649	0.7068 ± 0.09	4.54057E-07
	0.615				0.596		
	0.571				0.755		
	0.537				0.828		
	0.509				0.706		

**Figure 9:**
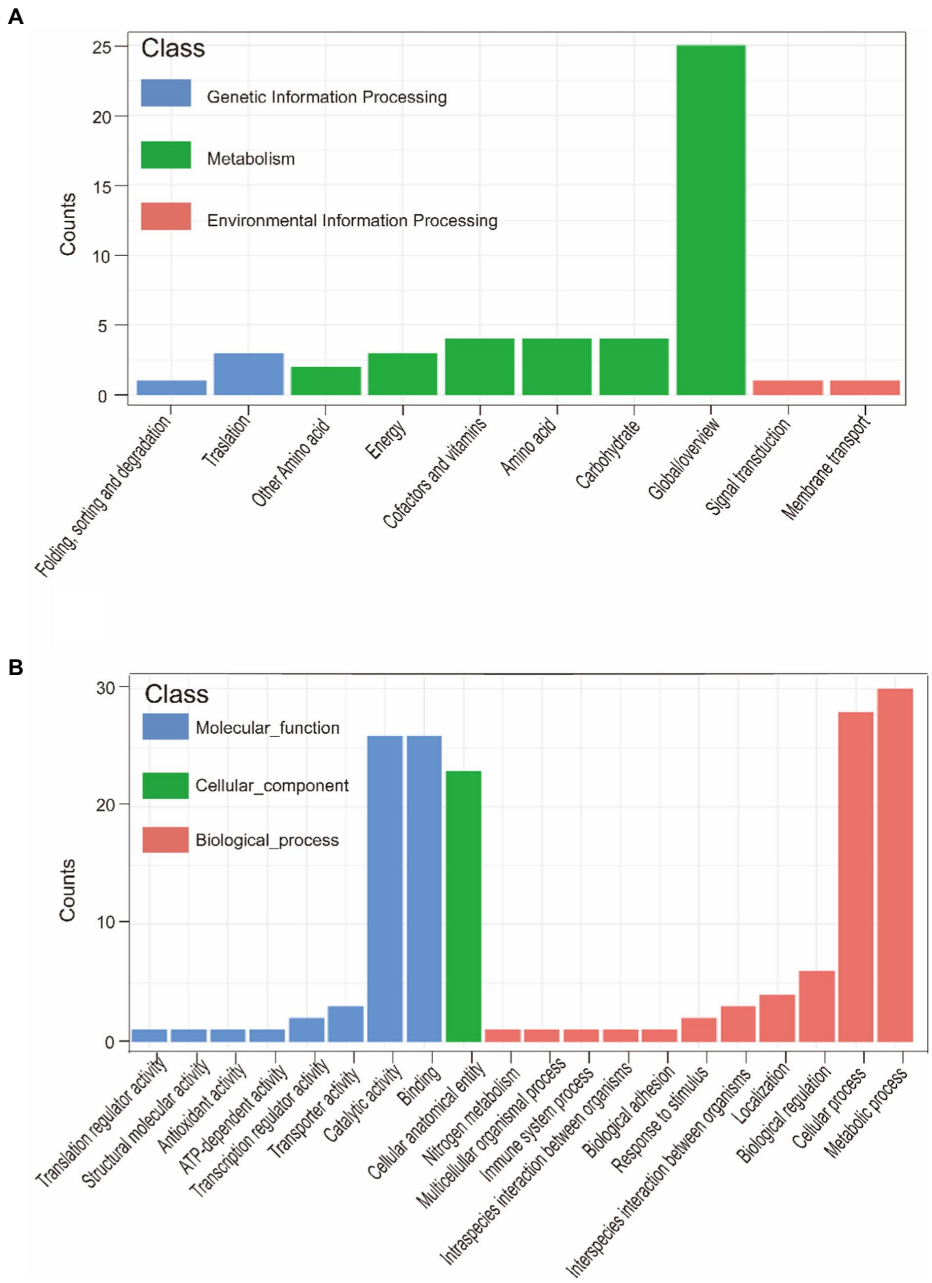
KEGG annotation of biofilm formation-associated genes **(A)**. GO annotation of biofilm formation-associated genes **(B)**.

Pan-GWAS results showed that 76 genes were associated with *G. parasuis* strong biofilm formation capacity, involved in signal pathways such as metabolism, genetic information processing, and environmental information processing (Figure 9). However, these genes associated with strong biofilm formation that could specifically identify the strong biofilm formation strains were not found. Metabolic pathways include carbohydrate, energy, amino acids, vitamins, etc. Importantly, the metabolism of nitrogen and nitrate may induce the denitrification process of the bacteria inside the biofilm in low dissolved oxygen conditions. Genetic information processing functional pathways focus on the translation process. Environmental information processing includes signal transduction and membrane transport. Furthermore, the functions of virulence factors concentrate on movement, immune modulation, nutritional metabolism, and adherence. For instance, the flagellum, which participates in movement and adherence, plays an essential role in the initial formation of biofilm and the dissipation process of the mature stage. In addition, lipoid A is modified by phosphoethanolamine transferase to reduce repulsion between the individuals *via* decreasing overall net negative charge of the bacterial outer membrane (Samantha and Vrielink, 2020). This way makes bacteria more easily clustered to form biofilm. Copper-transporting ATPases can regulate the copper balance and prevent copper poisoning while maintaining its nutrition (Migocka, 2015).

### 3.10. Antimicrobial susceptibility profiles

The MICs of six antimicrobial agents tested against 16 strains are summarized in Table 10. It can be seen from the results that 16 strains had varying degrees of resistance to aminoglycoside, β-lactam, tetracycline antibiotics. Overall, 16 strains showed the highest degree of the MIC of aminoglycoside antibiotics. This phenomenon also supported our results of CARD associated genes (Table 7). Namely, a large number of genes that against aminoglycoside antibiotics were found in cloud genes. Additionally, tetracycline also had a certain inhibitory effect on the isolated strain. A small number of genes involved in tetracycline also were found in shell and cloud genes. Of the six antibiotics tested, amoxicillin and ampicillin had the best bacteriostatic effect. And we did not find any genes related to β-lactam class in CARD associated genes. Hence, these results indicated the close association of antibiotic resistance phenotype and the composition of the CARD associated genes.

**Table 10:** Antibacterial activity test results.

Strains	MIC (μg/mL)					
	Amoxicillin	Ampicillin	Gentamicin	Kanamycin	Streptomycin	Tetracycline
GP 01	0.125	0.125	1	32	4	0.5
GP 02	0.062	0.062	4	32	8	2
GP 03	0.062	0.125	2	8	1	0.5
GP 04	0.062	0.5	2	8	32	16
GP 05	8	4	8	64	64	32
GP 06	0.031	0.031	4	8	0.5	0.125
GP 07	0.031	0.031	2	8	0.5	0.125
GP 08	0.125	0.062	8	16	1	1
GP 09	0.062	0.062	2	16	8	0.5
GP 10	0.062	0.125	4	8	2	0.5
GP 11	0.031	0.125	4	16	1	1
GP 12	0.5	0.5	8	32	16	8
GP 13	0.031	0.031	2	8	8	1
GP 14	1	0.5	1	32	32	16
GP 15	1	1	16	32	64	32
GP 16	0.062	0.062	2	8	0.5	0.5

### 3.11. Cell adhesion experiments

**Figure 10:**
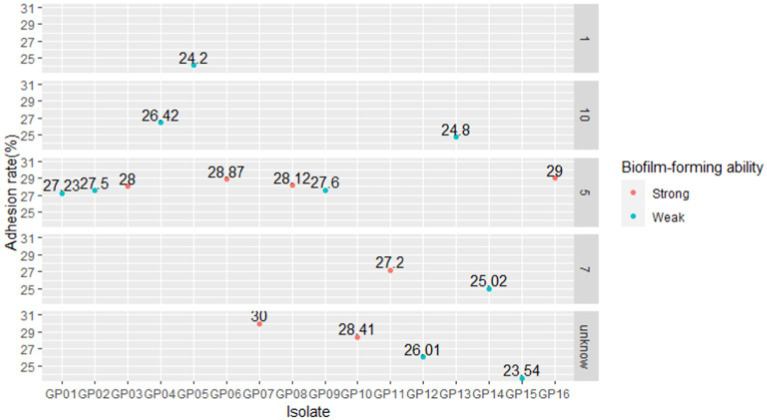
Comparison of adhesion rates of different isolates. We faceted the graphs according to the serotypes of the 16 isolates. According to the absorbance of different isolates (Table 9), with an OD630 value threshold of 0.5, it was divided into strong biofilm-forming strains and weak biofilm-forming strains, and the color was distinguished.

The results of cell adhesion experiments are shown in Figure 10. In a previous study, we classified 1,5,7,10 serotypes of 16 strains as virulent strains in order to search for genes associated with virulence (Table 2). In cell adhesion experiments, we also used this approach to differentiate between the virulence of the 16 strains. Our results showed that strains of serotype 5 exhibited higher adhesion rates in virulent strains. However, the reasons for the difference in adhesion rates between the different serotype strains were still unknown. Although the GP 07 shows the highest adhesion rate in 16 strains, the adhesion rate between different strains is large in the unknown serum type (Figure 10). Confusingly, we did not find a clear relationship between virulence and adhesion rate. However, *via* differentiating the biofilm-forming capacity of the 16 strains, we seem to find that there may be a positive correlation between the adhesion ability of *G. parasuis* and the ability to form biofilms (Table 9, Figure 10). Namely, the adhesion rates of these strong biofilm-forming strains were higher than those with weak biofilm-forming ability.

## 4. Discussion

*Glaesserella parasuis* is rich in genetic diversity, with a strong ability to acquire foreign genes. The phenotype (virulence, biofilm formation) varies greatly between strains. Pan-GWAS is an effective method for studying traits and gene associations (Gori et al., 2020). In this study, *G. parasuis* pan-genome analysis showed that only 390 genes were shared by different individuals, namely core genes. The core gene is a basic skeleton for supporting remainder of the genome, rather than the minimal genome necessary for bacterial survival. If the definition of the core is enlarged (including genes missing only in small parts of the genome), core genomes consist of 1,133 genes (including core genes and soft genes). These genes were present in at least 99% of the sample genomes. The number of core genome also were consistent with previous study (Howell et al., 2014). Our result indicated that *G. parasuis* has opening pan-genome, and the number of core genome did not change significantly with the increase in the number of strains. In contrast, genes in some strains and specific genes only remaining in a single strain constitute an accessory genome, including 7,752 genes.

Core genes not only included all COG classifications, but also majorly focused on basic and necessary biological functions in comparison to accessory genome, such as energy production and conversion, essential substances transport and metabolism, translation and so on. Importantly, cytoskeleton was found only in the core genes. Bacterial cytoskeleton structures are a filamentous structure which based on polymers of a single class of protein (Shih and Rothfield, 2006). Bacterial cytoskeleton plays an essential role in cell division, cell polarity, cell shape regulation, plasmid partition, and other functions (Michie and Löwe, 2006). Presumably, the core genes may play a key role in maintaining shape, multiplication and heterogeneous resistance of bacteria. Moreover, lethal factors were not found in core genes. These cases reflected that the essence of core genes is to control bacterial normal morphology, reproduction and to perform basic biological functions. In addition, the accessory genome also found genes that can participate in *G. parasuis* basic biological process. Presumably, the accessory genome may have an alternative function to maintain part of the biological process of bacterial when damaged core genes.

Pathogenic genes were largely found in accessory genome. These genes participated in bacterial resistance, colonization and invasion. Interestingly, the strains with these genes are mainly isolated from the pericardium and heart blood, rather than the joints and nasal cavity. Our results were consistent with previous studies (Yu et al., 2014; Van et al., 2019). Namely, the avirulent strains mainly adhere to and colonize the upper respiratory tract. Therefore, accessory genome was presumed to be the main source of bacterial pathogenicity.

Interestingly, a proportion of the virulence factors were found in core genes and had various pathogenic mechanisms. Thus, even *G. parasuis* strains considered avirulent are equally potentially pathogenic, and these strains should be included in the prevention and control scope. Virulence factors of the accessary genome major focus on biofilm forming, adherence, immune modulation, invasion, capsule etc. These functions confer the bacteria a greater resistance to the host immune system and contribute to triggering further infection.

Additionally, antimicrobial resistance (AMR) genes were widespread in accessory genomes rather than core genes. The horizontal transfer of AMR genes was the major way bacteria obtain drug resistance in previous studies (Kung et al., 2010; Croll and McDonald, 2012). Presumably, with the increase of genome number, AMR genes will be widespread in *G. parasuis* strains, due to *G. parasuis* had an open pan-genome. Moreover, combined with antibacterial activity test results, it was found that *G. parasuis* has strong resistance to aminoglycoside antibiotics and has a tendency to tolerate tetracycline antibiotics. Presumably, the acquisition and loss of AMR genes did affect the antibiotic phenotype of *G. parasuis* to certain extent. Hence, the misuse of antibiotics to treat *G. parasuis* infections likely contributed to the development and spread of antibiotic resistance in *G. parasuis*.

Although 142 genes were linked to *G. parasuis* hypervirulence traits in this study, the virulence factors that could specifically identify the hypervirulent strains were not found. However, some virulence genes associated with traits were found *via* Pan-GWAS. For instance, the glycosyltransferase that participates in LPS synthesis and modification is essential for adherence and invasion in the pig host. The glycosyltransferase has been shown to inactivate host proteins *via* glycosylation. This process will disturb signal transduction and immune response to achieve immune evasion (DeAngelis, 2002; Zhang et al., 2014). The *IbeA* invasion protein, mediating bacteria in crossing the blood–brain barrier, is essential for invasion (Huang et al., 1995). Colibacillus lacking the *IbeA* protein are unable to invade cerebral microvascular endothelial cells (Huang et al., 2001). In some acute cases, central nervous system symptoms caused by *G. parasuis* infection may be implicated in invasion protein represented by *IbaA*. Moreover, *IbeA* was presumed to locate in the extracellular membrane. And it can be used as a potential vaccine antigen target. Trimeric autotransporters (VtaAs) are only found in virulence strains and play an essential role in adherence and anti-phagocytosis (Laarmann et al., 2002; Meng et al., 2006). Our study also supports this view. Virulence-associated VtaAs can interfere with the phagocytosis of the host leukocytes and promise vaccine candidates. Sialyltransferase (lsgB) is involved in sialic acid utilization (Bouchet et al., 2003). Huan Wang found that *G. parasuis* strains lacking the lsgB are unable to effectively invade porcine iliac artery endothelial cells and porcine kidney epithelial cells due to the decreased autoaggregation ability of bacteria (Wang et al., 2021). Moreover, LOS mediated by lsgB is associated with resistance to the bactericidal effects of complement in the blood (Lewis et al., 2012). These data suggest the difference between avirulent and virulent strains is mainly reflected in entering the host, escaping host defense, bacteria multiplication, and damaging tissues.

Biofilm forming is critical for a successful bacteria survival and infection host. Especially, biofilm forming had been shown to be associated with virulence, antibiotic resistance and genetic typing (Dufour et al., 2010). In this study, although 76 genes were found that are associated with strong biofilm-forming ability, the strains with a strong biofilm-forming phenotype showed differences in the composition of these 76 genes. Moreover, some of the 76 genes also were found in some strains with a weak biofilm-forming phenotype. Presumably, the strain biofilm phenotype is the dynamic result of multiple gene regulation. Even between strains of the same serotype, the ability to form biofilms varies greatly. This may be due to the fact that *G. parasuis* has an open pan-genome, and isolates in different regions differ in the composition of the accessory genome, which in turn affects the biofilm phenotype. In addition, these 76 genes involved in signaling pathways such as metabolism, genetic information processing, and environmental information processing. Nitrogen and nitrate metabolism associated with denitrification play an important role in anoxia conditions (Richardson and Watmough, 1999). Bacterial located in a hypoxic microenvironment within a biofilm can utilize nitrate as the electron acceptors to complete cellular respiration. Furthermore, complex dynamic and metabolic heterogeneity were found in biofilm in the previous study (Flemming et al., 2016; Karygianni et al., 2020). Anaerobic metabolic processes may have local effects on the microenvironment *via* by-products to maintain biofilm homeostasis. Interestingly, pathways related to ethylbenzene degradation, naphthalene degradation, and degradation of chloroalkene were found in the accessory genome (Rabus and Widdel, 1995). These data showed that certain strains might obtain fitness advantages under specific environmental conditions. For example, certain *G. parasuis* strains can survive long-term and cause persistent infection in anaerobic environments such as host tissue or within the biofilm (Dar et al., 2021). Lipid A phosphoethanolamine transferase, coded by *opgE_2* gene, can reduce the overall net-negative charge of the outer membrane of some gram-negative bacteria *via* modification of lipid A. This process will confer resistance to polymyxin (Samantha and Vrielink, 2020). Hence, our results also supported an intimate connection between biofilm presence and antibiotic resistance (Mah and O’Toole, 2001). Furthermore, the decrease of net-negative charge is putatively helpful to abate repulsion between bacteria individuals. Bacteria can autoaggregate *via* this way. Autoaggregation and microcolony formation are among the first steps in building a biofilm (Trunk et al., 2018). Thus, lipid A phosphoethanolamine transferase may be a potential molecular target for antimicrobial agents and vaccine design.

Generally, the high-biofilm production phenotype linked to virulence is blurry (Jin et al., 2006; Bello-Ortí et al., 2014). In the present study, virulence and biofilm forming may have potential relation. Our results showed that the same protein products were found in strong virulence association genes and strong biofilm formation association genes, including transferrin-binding protein two and fibrinogen binding M-like protein (SiM protein) (Table 8 and Table 11). Transferrin-binding protein plays an essential role in iron acquisition (Moraes et al., 2009; Calmettes et al., 2012). Pathogenic bacteria can use high-affinity iron uptake systems, such as transferrin-binding proteins, to capture the iron of the host (del Río et al., 2005; Curran et al., 2015). Similarly, transferrin-binding protein expression is beneficial to maintaining bacteria survival under low iron conditions, such as within biofilm. SiM protein is the dominant virulence factor in some streptococcal species. SiM protein can confer bacterial resistance to phagocytosis *via* binding fibrinogen (Baiano et al., 2008). Expression of SiM protein in virulence strains may block the deposition of complement on bacterial surface. This process will confer bacterial evasion of phagocytosis and multiply within the host (Geyer and Schmidt, 2000). Additionally, SiM proteins were found to play a role in bacterial autoaggregation (Trunk et al., 2018). On the one hand, autoaggregation may represent an additional virulence mechanism. Virulence is enhanced by the formation of aggregates (Bieber et al., 1998). On the other hand, autoaggregation is more beneficial for bacterial adhesion to the tissue surfaces to form the biofilm (Sherlock et al., 2005). Hence, we postulated that these two products may become a marker of the relationship between virulence and biofilm forming.

**Table 11:** VFDB annotation of strong biofilm formation-associated genes.

Gene	Production	Function
group_979	RNA polymerase factor	Flagellum mediates movement
group_320	M-related protein Enn	Immune modulation
group_421	PE domain-containing protein	Immune modulation
group_1171	Nitrate reductase	Nutritional/Metabolic
opgE_2	Phosphoethanolamine transferase	Nutritional/Metabolic
yiaJ	DNA-binding transcriptional repressor	Nutritional/Metabolic
group_829	Transferrin-binding protein 2 precursor	Nutritional/Metabolic
copA	Copper-translocating P-type ATPase	Nutritional/Metabolic
bioD	Dethiobiotin synthetase	Nutritional/Metabolic
bioC	Biotin synthesis protein	Nutritional/Metabolic
fucO	Adhesion protein Lap	Adherence
group_279	Surface-exposed protein, autotransporter	Adherence
group_2798	Elongation factor Tu	Adherence
group_4247	SiM protein	Adherence

Generally, some virulence factors play a crucial role in the adhesion and colonization of bacteria. In our study, some virulence factors that favor bacterial adhesion also were found (Table 6). For example, the fimbrial protein, encoded by the *PilE* gene, has been proven to mediate bacterial adherence to mucosal epithelia (Xu et al., 2011). LOS is not only involved in immune regulation, but also acts as an adhesion factor in *G. parasuis*-triggered meningitis (Wang et al., 2021). However, in cell adhesion experiments, we did not observe certain exact relationship between the different serotypes and cell adhesion rates. This may be due to annotation to adherent virulence factors predominantly present in the accessory genome. Even with strains of the same serotype, there are still large differences in genetic composition, which leads to differences in adhesion ability between strains of the same serotype (Figure 10). Although the biofilm formation process is extremely complex, initial adhesion is necessary for bacteria to form biofilm (Arciola et al., 2018). Our results found that a correlation between adhesion and biofilm. Namely, the cell adhesion ability of the *G. parasuis* can be directly linked to its biofilm-forming ability. However, limited to only 16 strains, this connection still needs to be verified in a large number of samples.

Our analysis has some limitations. First, we were confined to the current publicly available *G. parasuis* genomes retrieved from NCBI (a total of 106 isolates) and 16 genomes from CNSA. Second, our study was limited to the biofilm formation process *in vitro*. In fact, biofilm formation is a complex process engaging various steps and physiologically diverse cellular states. The expression of some genes may differ during the biofilm formation process *in vitro* or vivo. Third, as our analysis is confined to putative protein in the database, further experiments will be needed to verify these supposed genes linked to the traits. Finally, due to the small sample size, experiments using gene-knockout may be useful for evaluating the hypothesis that virulence and biofilms may have potential links.

In conclusion, we have shown the characteristic differences in the core genome and the accessory genome of *G. parasuis*. Moreover, we screened out 142 genes with an association with strong virulence. This may establish the basis for the search for new virulence factors. In biofilm formation, multiple genes are involved in prophase adhesion, and autoaggregation processes to influence biofilm formation by regulating metabolism, and may contribute to the pathogenicity of *G. parasuis* pathogenicity. Additionally, we speculated that virulence and biofilms may have potential links. Finally, we assumed a positive correlation between the adhesion rate of *G. parasuis* on the cell and biofilm-forming ability of *G. parasuis*.

## Data availability statement

The datasets presented in this study can be found in online repositories. The names of the repository/repositories and accession number(s) can be found in the article/Supplementary material.

## Author contributions

YZ, MR, and DJ: conceptualization. YZ and DJ: software. YZ and MR: methodology. YZ, MR, and YY: formal analysis. YZ and GZ: investigation. ZY, YL, and YW: resources. DJ and MR: data curation. YZ and AL: writing—original draft preparation. DJ, ZY, MR, and YW: writing—review and editing. YZ and YW: visualization. MR and YW: supervision. YZ, MR, ZY, and XY: project administration. YL and YW: funding acquisition. All authors have read and agreed to the published version of the manuscript.

## Funding

This project was supported by the Sichuan Province Science and Technology Planning Program (2021ZDZX0010, 2021YJ0270, 2020YJ0345, and 2021YFSY0005), Provincial Natural Science Foundation of Sichuan (2023NSFSC1216), China Postdoctoral Science Foundation (2022M722300), and the Hong Kong Scholars Program 2022, China Postdoctoral Science Foundation (XJ2022047).

## Conflict of interest

The authors declare that the research was conducted in the absence of any commercial or financial relationships that could be construed as a potential conflict of interest.

## Publisher’s note

All claims expressed in this article are solely those of the authors and do not necessarily represent those of their affiliated organizations, or those of the publisher, the editors and the reviewers. Any product that may be evaluated in this article, or claim that may be made by its manufacturer, is not guaranteed or endorsed by the publisher.

## Footnotes

^1^ ftp://ftp.ncbi.nih.gov/genomes/all/

^2^ https://db.cngb.org/

^3^https://github.com/tseemann/prokka, accessed on 5 September 2021.

^4^https://github.com/sanger-pathogens/Roary, accessed on 6 October 2021.

^5^https://github.com/AdmiralenOla/Scoary, accessed on 8 September 2021.
