# Supplementary figures and images for: Pan-genome wide association study of Glaesserella parasuis highlights genes associated with virulence and biofilm formation
Source: Front Microbiol. 2023 Apr 17;14:1160433. doi: 10.3389/fmicb.2023.1160433 (PMC10149723; doi:10.3389/fmicb.2023.1160433)

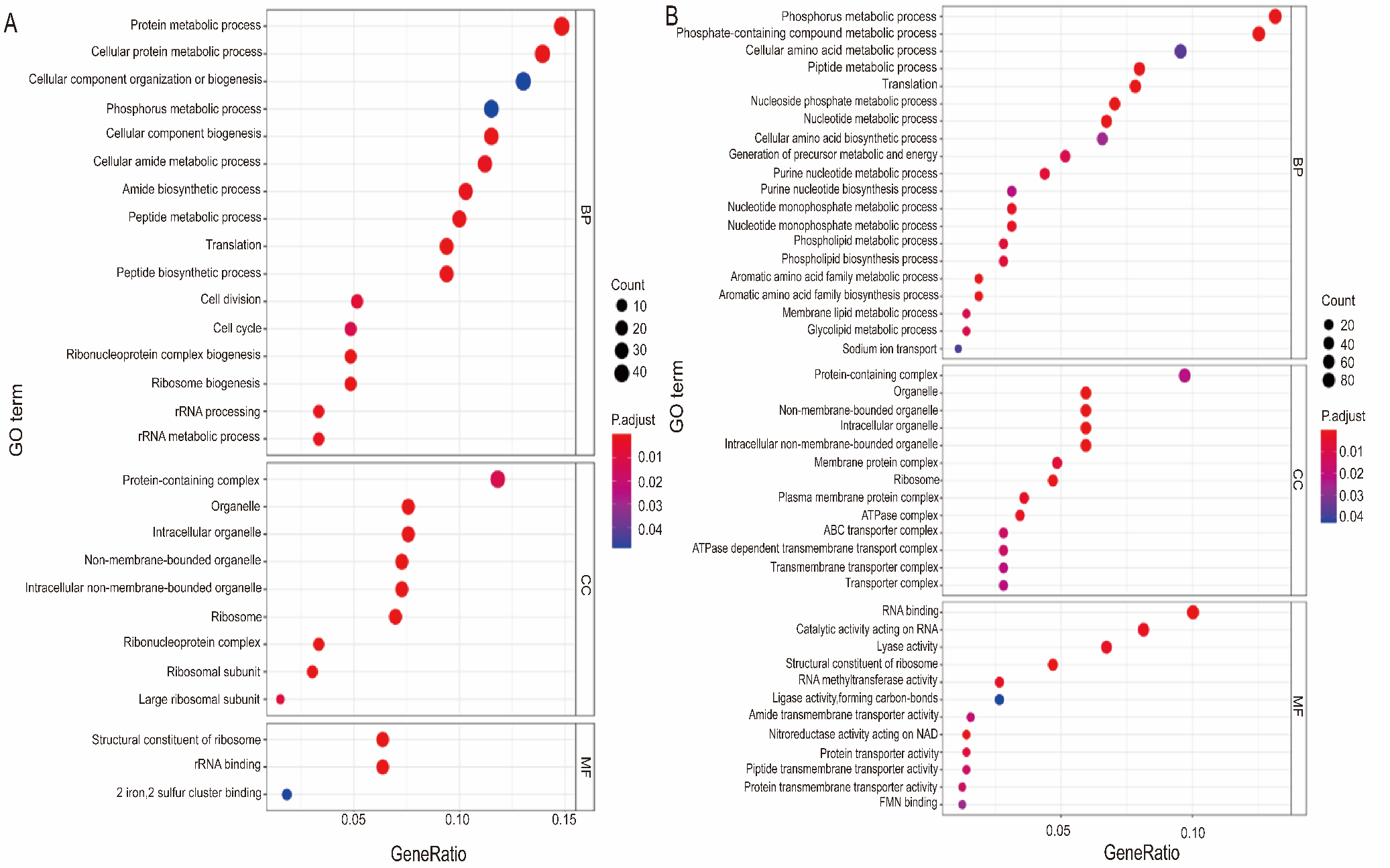


**Supplementary Figure 1.** GO enrichment of the core genome.

Supplement: Supplementary file 6 [file Data_Sheet_1.docx]
